# Subtalar Coalition: A Case Report

**DOI:** 10.5704/MOJ.1311.011

**Published:** 2013-11

**Authors:** CG Chua, EJ Yeap, M Yazid

**Affiliations:** Department of Orthopaedics and Traumatology, Hospital Tuanku Fauziah, Kangar, Perlis; Perlis Clinical Research Centre, Hospital Tuanku Fauziah, Kangar, Perlis; Department of Orthopaedics and Traumatology, Hospital Tuanku Fauziah, Kangar, Perlis

## Abstract

Subtalar coalition is an uncommon condition that usually manifests in early adolescence(1). Frequently, this condition is missed. Delayed diagnosis may result in osteoarthritis requiring triple arthrodesis. Here, we report two patients with subtalar coalition. The first patient is a 12 year old boy who presented with right ankle pain for one year and was treated with excision of the coalition and bone wax insertion at the excision site. We followed up the patient for two years and the result was excellent with full range of movement of his right ankle and subtalar joint attained within two months. He returned to athletic activity by six months and was discharged with no complications after two years. The second patient is a 15 year old girl who presented with bilateral ankle pain and swelling for three years and was treated with excision of the coalition and subtalar interpositional arthroplasty bilaterally. She defaulted follow up after seven months as she was very satisfied with the result. We wish to highlight this condition which may be misdiagnosed as flexible flat foot or ankle sprain.

**Key Words:**

Subtalar coalition, excision of coalition, bone wax insertion, subtalar interpositional arthroplasty

## Introduction

Talo-calcaneal coalition is an abnormal bridge between the talus and calcaneum, causing hindfoot pain, bony swelling and restriction of subtalar movement. The abnormal connection can be bony, cartilaginous or fibrous in nature with reported incidence ranging from 1 to 12%^1^. Symptoms usually manifest in early adolescence when the bridge starts to ossify and the diagnosis is made clinically and confirmed with CT scan if osseous fusion, or with MRI if fibrous or cartilaginous fusion .We report two typical cases of subtalar coalition in two adolescents with similar presentations .This case report stresses the importance of clinical suspicion in an adolescent who presents with ankle pain and restricted motion with flat feet. A high index of suspicion is required so as not to miss this easily treatable condition.

## Case Report

A 12 year old Siamese boy who was active in sports presented to our clinic with the complaint of pain and swelling over the medial aspect of his right ankle for a year. The swelling gradually increased in size and the pain worsened during walking and running. The pain was described as throbbing in nature and located at the posterior aspect of the right ankle. Patient also claimed that he had difficulty in moving the ankle sideways. He had no previous history of trauma to his right ankle.

**Fig. 1:**
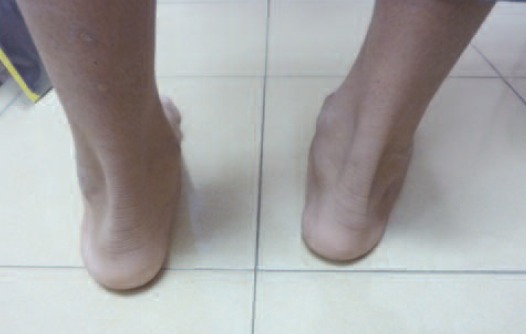
: Swelling below the right medial malleolus.

Physical examination revealed a 3 x 4 cm swelling below the right medial malleolus, tender on palpation, bony hard in consistency, surface, non mobile and the skin overlying it was normal.(Fig 1) There was pain on inversion and eversion of the ankle and the range of movement of subtalar joint was restricted.

**Fig. 2:**
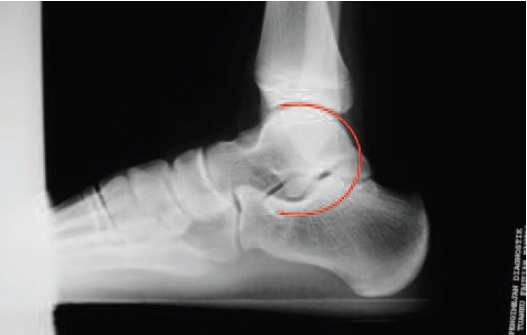
: Subtle C sign on right ankle lateral view X-ray.

**Fig. 3:**
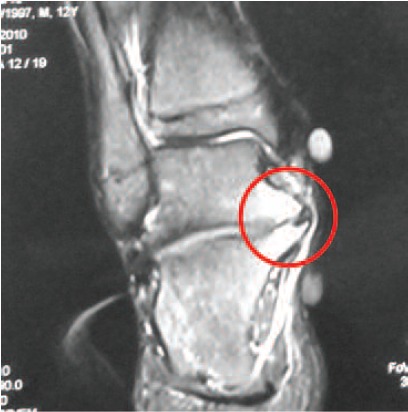
: Oedema within the right calcaneum and talus with early osteophyte formation on MRI.

Radiographs revealed a subtle C sign.(Fig 2) He was then planned for MRI of the right ankle and it revealed oedema within the right calcaneum and talus with early osteophyte formation and this could be due to the chronic trauma.(Fig 3) He was then diagnosed to have right talo-calcaneal coalition and scheduled for excision of the talo-calcaneal coalition with bone wax insertion.

We proceeded with the medial approach, just below the medial malleolus. Intra-operatively, we noted the right talocalcaneal joint space was immobile. The coalition was resected and bone wax was inserted at the site of resection to prevent further bone formation. He was discharged on postop day 3 and allowed weight bearing as tolerated.

We followed up the patient by using the AOFAS scoring scale and the result was excellent. Pre-operatively, it was 76 and at six months post-op, it was 100. At two months postop, patient was able to fully bear weight without difficulty. Examination showed no pain on eversion and inversion of right ankle and the range of motion was full. After six months, he was already able to start playing football as before with no further swelling or pain. The last follow up was at two years post-op; he was asymptomatic and carrying out normal daily activities and sports with no difficulty. The radiograph film showed no osteoarthritic changes of his right subtalar joint.

The second patient was a 16-year old girl who presented with bilateral ankle pain and swelling for 4 years .The pain was dull in nature, aggravated by walking, relieved by rest, radiating to the heel and gradually worsening and thus limiting her daily activities. She also complained about difficulty walking on uneven surfaces. She had no previous history of trauma.

**Fig. 4:**
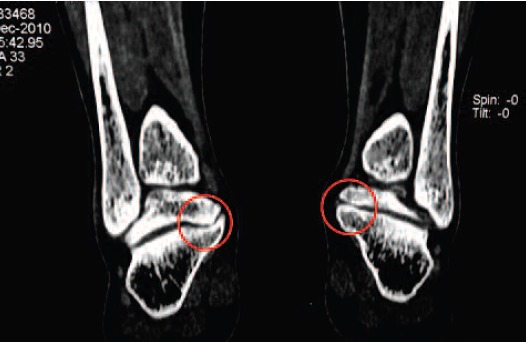
: CT scan of the 2nd patient showing prominent medial bony overgrowth with minimal joint space.

On examination, there were bilateral bony swellings at the postero-inferior aspect of medial malleolus which were tender. Skin overlying it was normal. The range of movements of ankles was full. Subtalar movement of inversion and eversion were associated with pain. CT scan The both revealed prominent bony overgrowth projecting medially from the sustentaculum tali and the medial part of the talus, appear to be impinging upon the adjacent flexor tendons.(Fig 4) She was diagnosed to have bilateral subtalar coalition and was scheduled for excision of coalition with subtalar inter-positional arthroplasty. We proceeded with the left foot first followed by the right foot which was done three months later. Intra-operatively, the medial approach was used and local fat graft was inserted into the subtalar joint space and the bone ends were covered with bone wax. These were applied for both sides. By six weeks, she was able to fully weight bear and returned to her daily routine. We only managed to follow her up until seven months post-op and she defaulted, presumably well. After 2 years, she was called back for reassessment and AOFAS scoring scale .We noted that she was very satisfied with the end result of the operation and had thus defaulted the follow up. The preoperative AOFAS scoring scale for the right side in this patient was 57 and left side was 58. After 2 years, the right side score was 72 whereas the left side was 69. Overall, the surgery almost totally met her expectations and she rated the end result of the surgery as very good.

## Discussion

Subtalar coalition can be congenital, as a consequence of autosomal dominant inheritance^1^. It also can be acquired by degenerative joint disease, inflammatory arthritis, infection and clubfoot deformity^1^. Talo-calcaneal coalition is usually fibrous or cartilaginous at birth then start to ossify during early adolescence. Patients with subtalar coalition usually present with hindfoot pain which is at the subtalar joint region. Both our patients presented with a throbbing type of hindfoot pain, beginning in early adolescence that worsened after continued activity. The onset of pain corresponded to the age of onset for congenital coalitions. Limited hindfoot mobility, especially the subtalar inversion and eversion is the most common physical examination findings in patients with talo-calcaneal coalition^2^. This finding is present in both our patients.

A large case series by Takakura et al demonstrated that a bony prominence inferior and posterior to the medial malleolus may be palpated in cases of talo-calcaneal coalition. These patients may also exhibit symptoms of tarsal tunnel syndrome, such as sensory disturbance in the sole of the foot and a positive Tinel’s test 2. Our patients did not demonstrate any symptoms of tarsal tunnel syndrome. X-ray may show the typical presence of a C-sign in the lateral view. (Fig 2) The C-sign is present when a continuous arc is seen on lateral radiographs between the medial cortex of the talus and the inferior cortex of the sustentaculum tali 3,4. This ‘C’ sign is a bony bridge between the talar dome and sustentaculum tali, in combination with a prominent inferior border of the sustentaculum tali. If it is not obvious, a CT scan or MRI may lead to the diagnosis 3. Crim et al determined that the most accurate radiographic sign for diagnosing talo-calcaneal coalition is the C-sign and talar beaking visualized on lateral views of the foot 4. Absent visibility of the subtalar facets, usually middle subtalar facet and a dysmorphic sustentaculum tali are also helpful in diagnosing talo-calcaneal coalition on lateral x-ray of the foot. However the accuracy of these signs can be confounded by the direction of the x-ray beam^4^.

There is lack of evidence to support the use of conservative management for subtalar coalition. Generally there are two options of surgical treatment, which is resection or fusion, with or without adjunctive reconstruction procedures3. Historically, tarsal coalitions have been treated with isolated or triple arthrodesis . However, as these patients were young with no evidence of arthritis, we proceeded with surgical excision of the coalition. Younger patients tend to fare better with resection. Yu G et al concluded that to achieve satisfactory outcomes for talo-calcaneal coalitions, a reasonable surgical procedure should be chosen according to the specific facet and complication^5^. The choice of interpositional graft ranges from fat and muscle to bone wax3. The outcomes for both patients showed significant improvement in their AOFAS scores. However, the outcome of surgery in the second patient was not as good as the first patient. This may be attributed to the bilateral involvement. Concurrent hindfoot valgus needs to be addressed, which was not present in our patients. Arthrodesis should be reserved for those with severe arthroses. We require longer follow up to monitor for this development.

In conclusion, clinical suspicion is very important in the diagnosis of talo-calcaneal coalition. With newer imaging modalities like CT and MRI, the diagnosis of subtalar coalition should not be missed as the outcome of early treatment is good. We recommend the use of surgical intervention in these young, active and symptomatic patients.
